# Lignin-Based Carbon-Fiber-Reinforced LVL Beams for Landscape Timber Structures

**DOI:** 10.3390/polym17152030

**Published:** 2025-07-25

**Authors:** Xuebo Li, Yuan Niu, Zhanpeng Jiang, Jiuyin Pang, Xiaoyi Niu

**Affiliations:** Key Laboratory of Wood Materials Science and Engineering, Beihua University, Jilin 132013, China; 13144328898@163.com (X.L.); niuyuan@nefu.edu.cn (Y.N.); jzp1578794094@163.com (Z.J.); pangjiuyin@163.com (J.P.)

**Keywords:** carbon fiber, lignin, timber structure, LVL beam

## Abstract

This study focuses on the development of lignin-based carbon-fiber-reinforced laminated veneer lumber (LVL) beams for garden timber structures, addressing wood shortages and environmental concerns. The research consisted of three main phases: the extraction and characterization of the lignin from corn stalks; the preparation and characterization of lignin-based carbon fibers; the fabrication and testing of reinforced LVL beams. Lignin was extracted from corn stalks using a deep eutectic solvent, followed by the preparation of lignin-based carbon fibers through electrospinning. These carbon fibers were integrated with poplar veneers to create reinforced LVL beams. The test results demonstrated significant improvements in mechanical properties, with the reinforced LVL beams exhibiting a 17% increase in elastic modulus and a 30% enhancement in flexural strength compared with conventional LVL beams. Notable improvements were also observed in tensile strength, compressive strength, and shear strength. This research provides a novel approach for producing high-value-added carbon fibers from agricultural waste, advancing the development of sustainable building materials.

## 1. Introduction

China’s garden architecture boasts a long and illustrious history, serving as a gem of global architectural art and representing humanity’s precious historical heritage. Within garden architecture, timber structures play an indispensable role [1,2,3,4]. Timber structures refer to engineering systems where wood serves as the primary load-bearing material. Owing to its advantages, such as its strong flexural resistance, compressive resistance, and seismic performance, timber has been widely utilized in China’s garden architecture. However, as these garden timber structures age, the inherent drawbacks of wood—including significant creep deformation, susceptibility to erosion, and aging—have become increasingly evident. During restoration, large quantities of timber are still required for reinforcement to preserve the original architectural integrity. Traditional reinforcement materials often fall short of desired outcomes due to limitations such as excessive weight and fail to meet the demands of an environmentally friendly society. Meanwhile, with the rapid development of the national economy, China’s demand for timber and its derived products has surged, accompanied by increasingly stringent quality requirements. As living standards improve, the demand for wooden products continues to rise. Since 2000, China has fully implemented the “Natural Forest Protection Program”, reducing the harvest of natural forest timber. The sharp decline in timber production has exacerbated the supply–demand imbalance [5,6,7,8]. To address these challenges, there is an urgent need to produce high-strength, stable-performance engineered wood products as substitutes for large-diameter structural logs, thereby alleviating timber shortages. Laminated veneer lumber (LVL), a type of structural engineered wood, represents one such solution.

Compared to natural solid wood, LVL exhibits superior characteristics. For instance, it outperforms natural timber in structural stability, mechanical properties, weather resistance, seismic damping performance, and economic efficiency. These advantages enable the principle of “enhancing the utilization of lower-grade and smaller-dimension timber”. Based on its applications, LVL can be categorized into structural and non-structural types. Internationally, particularly in Europe and North America, structural LVL has been extensively researched and utilized, often serving as load-bearing components such as trusses, floor joists, beams, columns, and load-bearing walls in timber structures. In China, however, the application of structural LVL remains in its early developmental stages. Structural LVL is widely employed in architectural load-bearing systems due to its exceptional stability and fire-resistant properties, which are superior to those of steel [9,10,11,12,13].

Beams are the primary load-bearing components in the upper timber frame of garden architecture, playing a critical role in these structures. LVL enables the fabrication of beams exceeding 20 m in length for architectural applications. Although LVL beams significantly reduce timber consumption compared to natural solid wood, the material usage remains substantial for large-span beams. For example, in Japan, open-web trusses have been employed in indoor stadiums with spans exceeding 100 m. However, LVL beams also exhibit drawbacks such as low density, small elastic modulus, pronounced directional anisotropy in stress distribution, and mechanical properties susceptible to creep effects. To address these challenges, carbon fiber composites have emerged as a promising solution [14,15,16]. Carbon fiber composites are advanced materials created by combining carbon fibers with other substrates. They offer exceptional comprehensive properties, including high strength, lightweight design, fatigue resistance, high-temperature tolerance, corrosion resistance, and operational safety. By integrating carbon fibers with LVL, the resulting carbon-fiber-reinforced LVL beams not only enhance overall performance and ensure structural stability but also reduce weight and conserve timber resources. Moreover, this approach aligns with sustainable development goals by minimizing environmental impact.

This study utilizes corn stalk powder as raw material to extract lignin and produce lignin-based carbon fibers, which are then combined with poplar veneers to fabricate LVL beams for garden timber structures. Through systematic research, the application potential of this novel composite material in garden timber architecture is explored, aiming to provide a theoretical foundation and technical support for advancing the sustainable development of timber structures [17,18,19,20,21,22].

## 2. Materials and Methods

### 2.1. Materials

Corn stalk powder (purchased from Surui Agricultural Products Deep Processing Co., Ltd., Suqian, China, acetone, deuterated dimethyl sulfoxide (DMSO-d6), polystyrene, potassium bromide (KBr), tetrahydrofuran (THF), oxalic acid dihydrate, polyacrylonitrile (PAN), N,N-dimethylformamide (DMF), lignin, choline chloride, zinc chloride (ZnCl_2_), and ethylene glycol (purchased from Shanghai Macklin Biochemical Technology Co., Ltd. (Shanghai, China)) were obtained. Poplar veneers were sourced from a local timber factory. Carbon fibers were self-prepared.

### 2.2. Extraction of Lignin

#### 2.2.1. Preparation of Low-Co-Melting Solvents (DES)

Choline chloride (hydrogen bond acceptor) and oxalic acid (hydrogen bond donor) were weighed at a 1:1 molar ratio, placed in a conical flask, and heated in an oil bath under constant temperature with continuous stirring until a colorless transparent liquid formed. Choline chloride and ethylene glycol were mixed at a 1:1 molar ratio and processed identically. Zinc chloride and ethylene glycol were combined at a 1:1 molar ratio and subjected to the same procedure.

#### 2.2.2. Draw

Using ChCl-EG as a deep eutectic solvent (DES), 1 g of corn straw powder was mixed with 20 g of DES and heated in an oil bath to 180 °C until slight black smoke was produced in the flask. Heating was then stopped, and the flask was removed and allowed to cool to room temperature for further processing. The deep brown solid–liquid mixture obtained from the previous step was added to acetone/water as an antisolvent and stirred at room temperature for 1 h.

The mixture was filtered using a vacuum filter, and the residue was washed three times with the same solvent. The filtered residue consisted of the undissolved solid remnants. The filtrate was poured into a shallow dish and placed in an oven for further heating to remove acetone. The concentrated solution was also filtered afterward to retain the solid, which was then placed in the oven for complete drying, yielding a lignin mass (LF). At this stage, the mass after treatment (CR) was 0.57 g, and LF was 0.16 g.

Next, using ChCl-H as DES, 1 g of corn straw powder was mixed with 20 g of DES and heated in an oil bath to 220 °C, with the subsequent procedure being the same as that described above. In this case, the mass after treatment (CR) was 0.41 g, and LF was 0.26 g.

Lastly, using ZnCl_2_-EG as DES, 1 g of corn straw powder was mixed with 20 g of DES and heated in an oil bath to 220 °C, with the following operations being the same as before. In this case, the mass after treatment (CR) was 0.69 g, and the LF was 0.13 g.

### 2.3. Preparation of Lignin-Based Carbon Fiber

Lignin-based carbon fibers were prepared by electrospinning method, with the following steps: the spinning solution was prepared; the electrospinning mechanism was conducted to prepare the precursor fibers; the precursor fibers were pre-oxidized and carbonized; the precursor fibers and carbon fibers were tested and characterized.

#### 2.3.1. Preparation of Electrospinning Solution

**Figure 1:**
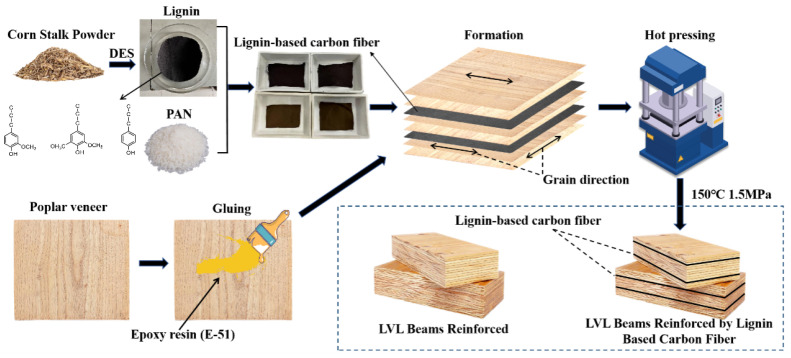
Diagrammatic drawing of preparation of spinning solution.

The extracted corn straw lignin was blended with DMF to prepare a spinning solution with a mass fraction of 20%. The mass ratios of lignin/PAN were set at 2:8, 3:7, 4:6, and 5:5. Four groups of samples were labeled as PL-28, PL-37, and PL-46. In this labeling, L stands for corn straw lignin and P stands for PAN. The specific steps were as follows: 16 g of DMF solution was added to a container, and PAN powder was added in small amounts multiple times to a beaker while stirring at 80 °C until PAN was completely dissolved; then, corn straw lignin was added in small amounts multiple times to the beaker until it was fully dissolved, resulting in a brown viscous solution [23,24,25,26]; the spinning solution was prepared using the method of mixing the extracted lignin with PAN, as illustrated in Figure 1.

#### 2.3.2. Preparation of Fiber Membrane by Electrospinning

The specific steps for the preparation of fiber membrane by electrospinning were as follows: prepared spinning solution was transferred into a 10 mL syringe with a needle aperture of 19 G; this was placed into a high-voltage electrospinning machine; the distance between the needle and the receiver (diameter 10 cm, length 25 cm, speed 400 r·min) was set to 170 mm, and a layer of aluminum foil was wrapped on the surface of the receiver to collect the fiber felt; the voltage of the electrospinning machine was set to 14 kV (12 kV, −2 kV); the injection speed of the syringe was set to 0.05 mL/h.

#### 2.3.3. Pre-Oxidation and Carbonization

The pre-oxidation treatment was conducted as follows: the raw fiber slices were placed in a porcelain boat and then into a muffle furnace; they were heated up to 260 °C at a rate of 1 °C/min in an air atmosphere; the temperature was maintained for 90 min, and then left to naturally cool down. The carbonization treatment was conducted as follows: the pre-oxidized slices were transferred to a tube furnace and heated at a rate of 5 °C/min to 800 °C under a nitrogen atmosphere; after holding for 90 min, the temperature naturally decreased [23].

### 2.4. Preparation of LVL Beams for Garden Wooden Structures

Poplar veneer with dimensions of 1200 mm × 600 mm and a thickness of 2 mm was used; in addition, lignin-based carbon fiber was used as the raw material, and epoxy resin was used as the adhesive. Accordingly, 33 layers of laminated veneer lumber (LVL) beams were pressed. The bisphenol A epoxy resin was mixed with the epoxy resin curing agent T-31 in a ratio of 3:1 for use. The prepared adhesive was evenly applied to both sides of the core layer of the poplar veneer, employing a single-sided coating method with an application rate of 150 g/m^2^. The test hot-pressing temperature was 150 °C, and the hot-pressing pressure was 1.5 MPa, with longitudinal pressing being conducted during the assembly process. The pressed LVL beam was designated LVL-T.

A layer of the prepared epoxy resin adhesive was uniformly applied to the upper surface of the core layer of the third layer of the poplar plywood. A piece of carbon fiber cloth, cut to size, was laid out and then firmly rolled in one direction until the carbon fiber cloth was completely impregnated with the epoxy resin. Using the same method, another piece of cut carbon fiber cloth was bonded to the bottom surface of the core layer of the third layer of the poplar plywood. The assembly was alternated between poplar veneer and carbon fiber cloth, resulting in a total of 33 layers. The pressed carbon fiber LVL beam was designated LVL-CF.

According to the GB/T 36408-2018 [27] standard “Laminated Veneer Lumber” (hereinafter referred to as the standard), the prepared composite materials were subjected to moisture content measurement, adhesion delamination tests, modulus of elasticity and flexural strength tests, longitudinal tensile tests, longitudinal compressive tests, transverse parallel compressive tests, and longitudinal parallel shear tests [28,29,30,31,32].

### 2.5. Characterization

#### 2.5.1. Determination of Molecular Weight of Lignin (GPC)

The molecular weight of the lignin samples was determined using gel permeation chromatography (GPC, Waters 2414, USA) with polystyrene standards. The analysis was conducted at 30 °C using tetrahydrofuran (THF) as the mobile phase at a flow rate of 1.0 mL/min, with an injection volume of 20 μL.

#### 2.5.2. Fourier Transform Infrared Spectroscopy Detection (FT-IR)

Measures of 1 mg lignin sample and 150 mg potassium bromide were accurately weighed and ground in an agate mortar; these were pressed into tablets and placed on a sample table for testing. The spectral range used was 4000~500 cm^−1^.

#### 2.5.3. Thermogravimetric Analysis (TG)

The thermal stability of lignin was tested using a thermogravimetric analyzer (TGA, TA Q50, New Castle, DE, USA). A measure of 5–10 mg of the sample was weighed and placed flat on a platinum test plate. It was heated from room temperature to 700 °C at a rate of 10 °C/min in a nitrogen atmosphere (flow rate of 40 mL/min).

#### 2.5.4. Scanning Electron Microscopy (SEM)

The microstructures of the electrospun precursor fibers and carbon fibers were observed using scanning electron microscopy (SEM, Jeol JSM-7800F, Tokyo Metropolis, Japan).

#### 2.5.5. X-Ray Diffractometer (XRD)

The crystal structures of the carbon fiber samples were determined by X-ray diffraction (XRD, Dmax Rapid II, Tokyo Metropolis, Japan); analyses were conducted using a current of 35 mA, a voltage of 45 kV, a Cu target as the radiation source, and a scanning range of 10~80°.

#### 2.5.6. Raman Spectrometer (Raman)

The graphitized structure of carbon fiber was characterized by Raman spectroscopy (Raman, Renishaw in Via, Gloucestershire, UK), with specific detection parameters of laser 540 nm, power 5%, exposure time 10 s, scanning wavelength 500–4000 cm^−1^, and data processing to obtain the ID/IG ratio [25].

#### 2.5.7. Mechanical Property Testing of Raw Silk and Carbon Fiber

Tensile strength and Young’s modulus of carbon fibers were measured using a universal mechanical testing machine (Shimadzu Corporation, Kyoto Prefecture, Japan). Specimens (1 × 5 cm) were prepared with 2 × 2 cm cardboard attached to both ends to prevent slippage. A 100 N load cell and a crosshead speed of 1 mm/min were applied. Five replicates were tested for each group, and average values were reported.

#### 2.5.8. Determination of Moisture Content

According to GB/T 17657-2013 [33], “Test Methods for Physical and Chemical Properties of Artificial Boards and Decorative Artificial Boards”, six sets of test specimens were designed and produced, each containing six cubic test blocks with dimensions of 50 mm × 50 mm × 50 mm. Among them, specimens 1–3 were LVL-T and specimens 4–6 were LVL-CF.

#### 2.5.9. Determination of Impregnation Peeling Rate

The impregnation peel strengths of the laminated veneer lumber (LVL) beams and the lignin-based carbon-fiber-reinforced LVL beams were evaluated following the Type I test in GB/T 17657-2013. Specimens were boiled for 4 h, immersed in 63 °C hot water for 3 h, and dried at 63 °C for 3 h.

#### 2.5.10. Determination of Flexural Strength and Elastic Modulus

According to the specifications, the elastic modulus and then the flexural strength of the same specimen in this experiment were measured. The size of the test pieces was 1100 mm × 50 mm × 50 mm, with the length along the grain direction. Each group of test pieces was set with 5 pieces.

#### 2.5.11. Determination of Tensile Strength Along the Grain

According to the specifications, each group of specimens was set up with 5 pieces, with dimensions of 1100 mm × 50 mm × 50 mm, a cross-sectional width of 5 mm, and a loading fixture distance of 1.1 m.

#### 2.5.12. Determination of Longitudinal Compressive Strength

According to the specifications, each group of specimens was set up with 5 specimens. The size of the compression specimens along the grain is 125 mm × 50 mm × 50 mm, and the length is in the direction of the grain.

#### 2.5.13. Determination of Transverse Parallel Compressive Strength

Five specimens were set for each group, and the dimensions of the transverse parallel compression specimens were 150 mm × 50 mm × 50 mm. The width of the metal indenter was 50 mm and the length was 70 mm. The indenter was located at the center of the length direction of the specimen, and the loading direction was parallel to the direction of the adhesive layer of the specimen. The loading speed was set to 0.3 mm/min during the experiment, and the test was ended when the deformation reached 2.5 mm. The load was recorded when the specimen deformed by 1.0 mm.

#### 2.5.14. Determination of Parallel Shear Strength Along the Grain

This experiment was conducted according to the specifications, with 5 specimens in each group. The dimensions of the specimens were 63 mm × 5 mm × 50 mm × 50 mm, and the shear surface size was 50 mm × 50 mm. The test speed was 0.6 mm/min. The shear force was formed by applying pressure along the grain, and the shear surface of the specimen was parallel to the adhesive layer.

#### 2.5.15. Determination of Mechanical Performance Characteristic Values

The characteristic value of the elastic modulus of the specimen was determined to be the average of the elastic modulus test results of all specimens, accurate to 1000 MPa. The characteristic value of the transverse parallel compressive strength of the specimen was determined to be the average value of the transverse parallel compressive strength test results of all specimens at 1.00 mm deformation, accurate to 1 MPa. The characteristic values of the flexural strength, tensile strength along the grain, compressive strength along the grain, and lateral shear strength along the grain of the sample were taken as the percentile values with a statistical distribution confidence of 75% and 5%.

## 3. Results and Discussion

### 3.1. Extraction and Analysis of Lignin from Corn Stover

#### 3.1.1. Gel Chromatography (GPC)

Table 1 shows the weight average molecular weight (Mn), number average molecular weight (Mw), and degree of polymerization distribution (PD) of three groups of samples, with alkali lignin as the control group. According to the literature, the higher the molecular weight of lignin, the better the mechanical properties of the carbon fibers prepared from it [26]. From the table, it can be seen that the lignin extracted by DES, ChCl-H, has the highest molecular weight and a lower PDI value, indicating its high molecular uniformity. Therefore, DES was chosen, and the lignin extracted by ChCl-H was further characterized [28,29].

#### 3.1.2. Fourier Transform Infrared Spectroscopy Analysis (FT-IR)

**Figure 2:**
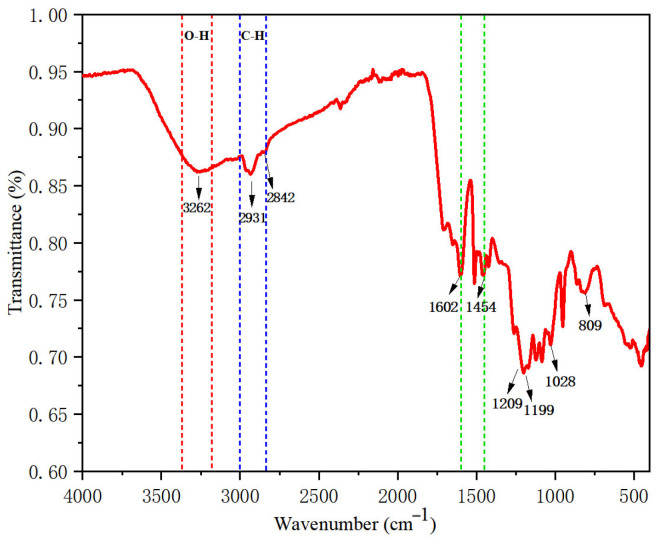
Infrared spectrum of lignin.

To investigate the structural changes of the lignin extracted from corn straw powder after treatment with the deep eutectic solvent (DES) ChCl-H, Fourier transform infrared (FT-IR) analysis was conducted on the extracted lignin samples, with the results shown in Figure 2. The analysis revealed that the absorption peaks at 1028 cm^−1^ and 809 cm^−1^ are characteristic of the para-hydroxy (H)-type structural units; the peak at 1199 cm^−1^ corresponds to the guaiacyl (G)-type structural units; the peak at 1602 cm^−1^ is representative of the syringyl (S) structural units. The range of 1602–1454 cm^−1^ consists of characteristic peaks due to the stretching vibrations of the aromatic ring backbone. The absorption peaks at 2931 cm^−1^ and 2842 cm^−1^ represent the stretching vibrations of C-H in the methylene groups [31]. A strong and broad peak at 3262 cm^−1^ indicates the stretching vibrations of O-H in hydroxyl groups. This infrared spectrum reflects the characteristic absorption of all functional groups present in the lignin molecule, indicating that this lignin belongs to the G/S/H-type lignin, which is characteristic of typical herbaceous lignin [32,34].

#### 3.1.3. Thermogravimetric Analysis (TG)

**Figure 3:**
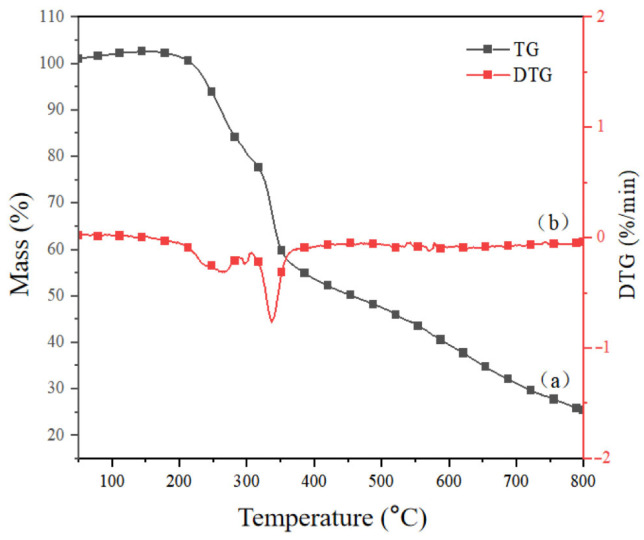
Thermal weight loss and weight loss rate curve of lignin in corn stover. a and b represent thermogravimetry and differential thermogravimetry respectively.

Figure 3 shows the TG (a) and DTG (b) curves of lignin in corn stover. The results showed that the weight loss at 50–200 °C was caused by the volatilization of residual solvents and other liquids in the lignin of corn stover; the main thermal degradation stage of the corn stover lignin occurred from 200 to 400 °C, during which carbohydrate components were decomposed into volatile gases (CO, CO_2_, CH_4_, etc.), and lignin reached its maximum weight loss rate at 336 °C; 400~600 °C reflects the breaking of aromatic rings and C-C bonds [35]. When the temperature exceeded 700 °C, there was about 30% solid residue, which reflects the ability of lignin to resist pyrolysis and shows that it has high thermal stability. It does not degrade even at high temperatures above 800 °C.

### 3.2. Characterization and Analysis of Lignin-Based Carbon Fiber

#### 3.2.1. Scanning Electron Microscopy Analysis (SEM)

**Figure 4:**
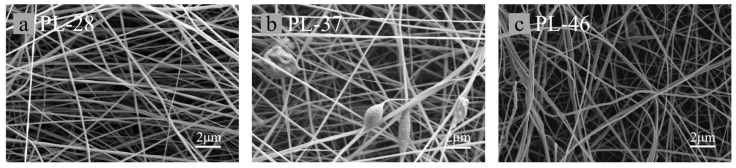
SEM images of electrospun lignin fiber membranes with different proportions.

**Figure 5:**
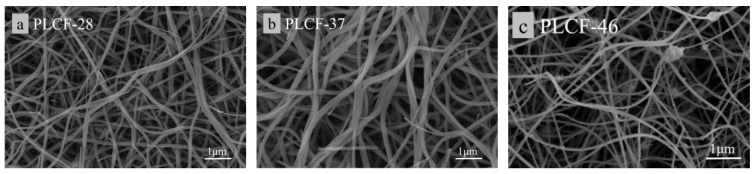
SEM images of lignin-based carbon fibers with different proportions.

Figure 4 shows the scanning electron microscopy (SEM) images of electrospun fibers with three different ratios: a for PL-28, b for PL-37, and c for PL-46. As shown in Figure 5, both a and b can produce relatively continuous fibers with uniform diameters and smooth surfaces. The diameter of a is slightly larger than those of b and c. This can be analyzed by noting that the addition of lignin during the preparation of the electrospinning solution reduced the viscosity of the spinning solution, which consequently led to a decreased ability to resist the driving forces from static electricity. From images b and c, it can be seen that, when the lignin proportion was 30%, the viscosity of the spinning solution decreased, resulting in a small amount of beading on the electrospun fibers. When the lignin proportion reached 40%, a significant amount of beading appeared on the surface of the electrospun fibers, which can greatly affect the mechanical properties of the carbon fibers formed after carbonization. The existing literature indicates that, when the mass ratio of lignin/PAN exceeds 3:7, a considerable amount of beading occurs in the fibers. In summary, corn straw lignin can partially replace PAN in the electrospinning process; however, its addition reduces the diameter of the electrospun fibers, indicating that lignin acts as a diluent during the electrospinning process [36].

Figure 5 shows the SEM images of carbon fibers. After carbonization, all fibers exhibit bending, with PLCF-28 and PLCF-37 maintaining good fiber morphology. However, PLCF-46 shows a significant number of fine nodules, which is believed to result from the excessive addition of lignin, leading to a polydisperse fiber morphology. Additionally, PLCF-46 displays slight melting and fusion, which can reduce the surface area of the fibers themselves and has a certain impact on their mechanical properties. Furthermore, on a macroscopic level, it can be observed that the surface area of the carbonized fibers is smaller compared to that of the pre-oxidized fibers. Similarly, on a microscopic level, the average fiber diameter of all fibers shows a trend of contraction, which is attributed to the large amounts of substances (such as H_2_O, CO, CO_2_, etc.) being released as gases during the high-temperature carbonization stage. The research indicates that larger diameters of carbon fibers are associated with poorer mechanical properties [37]. It can be observed that the diameter of PLCF-37 is significantly larger than that of PLCF-28, indicating that PLCF-28 exhibits the best mechanical properties among the three carbon fibers.

#### 3.2.2. X-Ray Diffraction Analysis (XRD)

**Figure 6:**
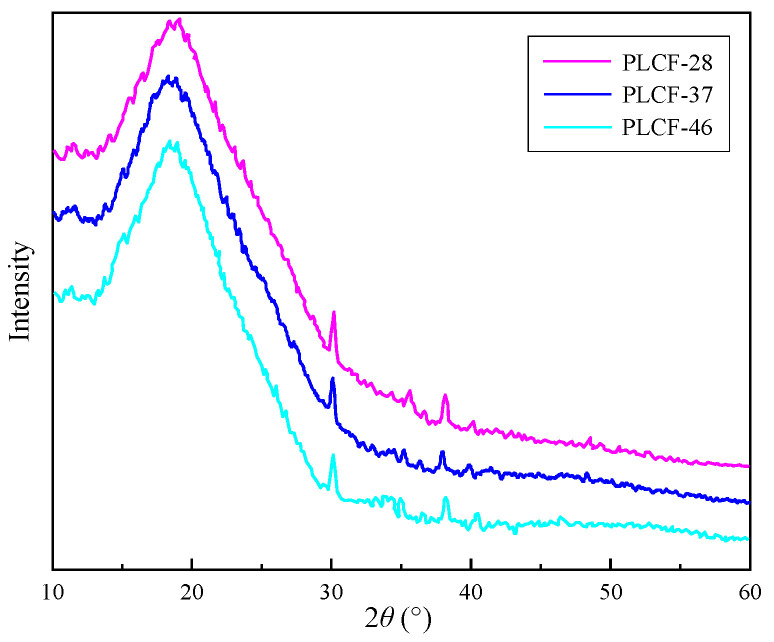
XRD pattern of carbon fibers with different proportions of lignin.

**Figure 7:**
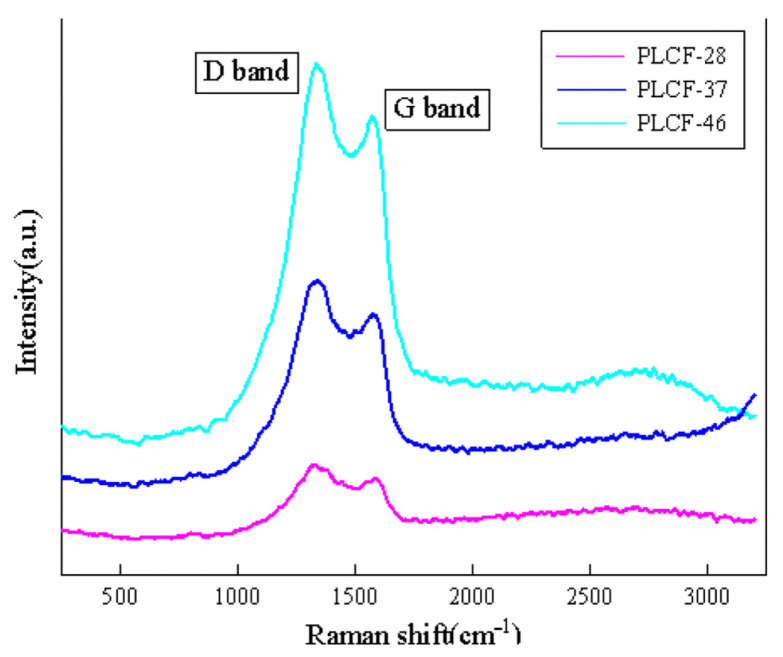
Raman spectra of PLCF-28, PLCF-37, and PLCF-46.

The mechanical properties of carbon fiber are positively correlated with its degree of graphitization. The thicker the continuous and ordered stacking of graphite microcrystals in carbon fibers, and the smaller the interlayer spacing, the higher the crystallinity, that is, the better the degree of graphitization, and the more significant the mechanical properties [38]. According to the analysis in Figure 6, the diffraction spectra of the three carbon fibers are basically consistent, indicating the stability of electrospun fibers when the lignin content is less than or equal to 40%. The diffraction peak at 2θ = 30° corresponds to the (002) crystal plane of carbon fiber graphite microcrystals, and the diffraction peak at 2θ = 38° corresponds to the (100) crystal plane of graphite crystals. There is a broad diffraction peak at around 15° to 23° for 2θ, indicating that the carbon structure in this lignin-based carbon fiber is amorphous, meaning that the carbon in this type of carbon fiber is amorphous carbon. Figure 7 shows the X-ray diffraction pattern of lignin-based carbon fibers obtained by heat treatment at 700 °C. 

#### 3.2.3. Raman Spectroscopy Analysis (Raman)

Raman spectroscopy is an effective tool for characterizing the degree of graphitization of carbon fibers. Figure 7 shows the Raman spectra of carbon fibers obtained by carbonizing lignin with different ratios at 800 °C. As shown in the figure, all three samples exhibit two distinct characteristic peaks around 1330 cm^−1^ (D peak) and 1570 cm^−1^ (G peak). The D peak returned due to the stretching vibration of sp3 hybridized bonds of unsaturated carbon atoms at the edge of the graphite layer; here, the incompleteness of the carbon material structure is reflected, that is, the carbon in the carbon fiber is amorphous carbon, which is consistent with the XRD analysis results. The G peak is the in-plane stretching vibration of sp2 hybridized bonds of carbon atoms in the graphite layer structure, representing the ideal graphitization structure [39]. The R value (ID/IG) is the intensity ratio of two characteristic peaks, which can reflect the degree of the graphitization of carbon fibers. A lower R value represents a more regular stacking of graphite layers and a higher degree of graphitization. Perform peak fitting on the spectrum to obtain the integrated R values of two peak areas. The R values of PLCF-28, PLCF-37, and PLCF-46 are 1.08, 1.12, and 1.14, respectively. The results indicate that the degree of graphitization of carbon fibers decreases with increasing lignin content.

#### 3.2.4. Testing and Analysis of Mechanical Properties of Raw Silk and Carbon Fiber

**Figure 8:**
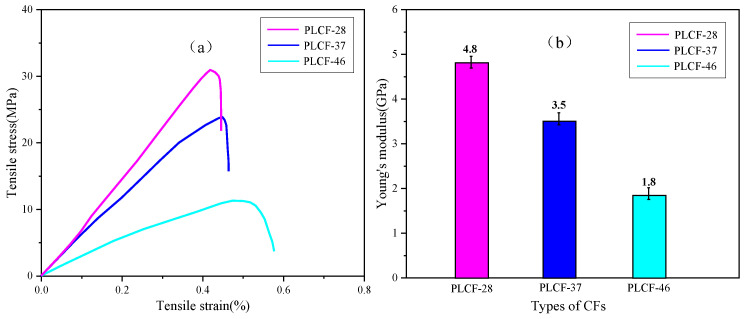
Mechanical properties of PLCF-28, PLCF-37, and PLCF-46: (**a**) the tensile strength–strain curve; (**b**) average Young’s modulus of the sample.

The two main indicators for measuring the mechanical properties of carbon fibers are tensile strength and Young’s modulus. Figure 8 presents the test and calculation results. Using the method described in Section 3.3.4, tensile strength and Young’s modulus were tested and calculated for four sample groups: PL-28, PL-37, PLCF-28, and PLCF-37. This was conducted in order to assess the effect of corn straw lignin on the mechanical properties of carbon fibers.

As seen in Figure 8a, the stress–strain relationships of the three types of carbon fibers are linear, exhibiting typical brittle fracture behavior of carbon fibers: the tensile strength of PLCF-28 is 30.4 MPa; PLCF-37 has a tensile strength of 22.9 MPa; PLCF-46 has a tensile strength of 11.5 MPa. Figure 8b shows that the Young’s modulus of PLCF-28 is 4.8 GPa, the Young’s modulus of PLCF-37 is 3.5 GPa, and the Young’s modulus of PLCF-46 is 1.8 GPa. Under similar processing conditions, the tensile strength of PAN-based carbon fibers can reach approximately 45 ± 5 MPa, indicating that the tensile strength of PLCF-28 is quite close, demonstrating that when the lignin/PAN ratio is 20:80, it can reduce the cost of carbon fibers while relatively ensuring their mechanical properties [40].

### 3.3. Testing of LVL Beams in Garden Wooden Structures

#### 3.3.1. Moisture Content Analysis

According to Table 2, the moisture content of LVL-T and LVL-CF is 12.3%, which meets the standard requirements.

#### 3.3.2. Analysis of Immersion Peel Strength

Table 3 shows the impregnation and delamination data of LVL-T and LVL-CF, indicating that the impregnation and delamination lengths of each specimen meet the standard requirements.

#### 3.3.3. Test Results and Analysis of Flexural Strength and Elastic Modulus

**Figure 9:**
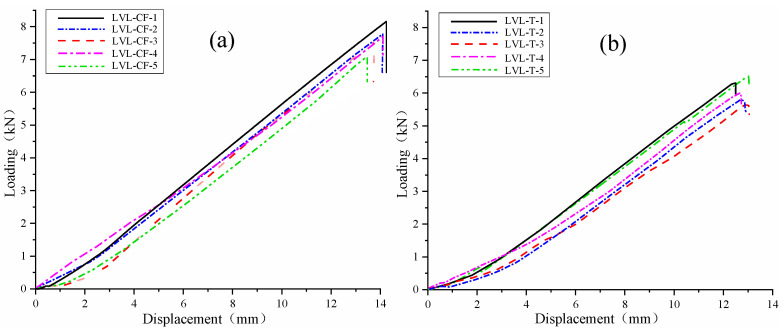
Bending strength load–displacement curve. (**a**) LVL-CF specimen bending test load–displacement curve. (**b**) LVL-T specimen bending test load–displacement curve.

The load–displacement curves of the bending test specimens LVL-CF (a) and LVL-T (b) are shown in Figure 9. The load–displacement curve indicates that, in the initial stage of applying load, (a) and (b), and in the initial stage of force, the deflection increases with the increase in load, and the component is in the elastic working stage. When the load reaches around 4.52 kN and 4.05 kN, the deformation of the component significantly increases, accompanied by continuous fracture sounds. As the displacement increases, the degree of bending of the specimen gradually increases. When the load reaches 6.45 kN and 5.38 kN, the ultimate state is reached, and the specimen fails with a loud noise. After unloading, the deformation of the components significantly recovered.

According to the data in Table 4 and Table 5, the average elastic moduli of a and b are 13,625.2 MPa and 11,682.3 MPa, respectively. Here, a reaches the strength grade of 13 × 10^−38^ while b reaches the strength grade of 10 × 10^−33^; the elastic modulus of a is 1.17 times that of b. The mechanical characteristic values of the bending strength of a and b are 49.28 MPa and 37.77 MPa, respectively. Here, a reaches the strength grade of 15 × 10^−45^, while b reaches the strength grade of 10 × 10^−33^; the bending strength of a is 1.30 times that of b. Comparing the experimental data with the standard values, with standard deviations of 2.37 and 2.31, the validity of the performance experimental data for the LVL-CF (a) and LVL-T (b) specimens is obtained, meeting the criteria for use as material parameters in finite element simulation and ensuring the effectiveness of the finite element simulation.

#### 3.3.4. Results and Analysis of Tensile Test Along the Grain

The longitudinal compressive test data of LVL-CF (a) and LVL-T (b) specimens are shown in Table 6 and Table 7. According to the data in the table, the characteristic value of the tensile strength of a along the grain is 64.96 MPa, reaching the strength level of 15 × 10^−45^; the characteristic value of the longitudinal compressive strength of b is 41.80 MPa, which also reaches the strength grade of 15 × 10^−45^. The longitudinal compressive strength of a is 1.55 times that of b.

#### 3.3.5. Results and Analysis of Longitudinal Compression Test

**Figure 10:**
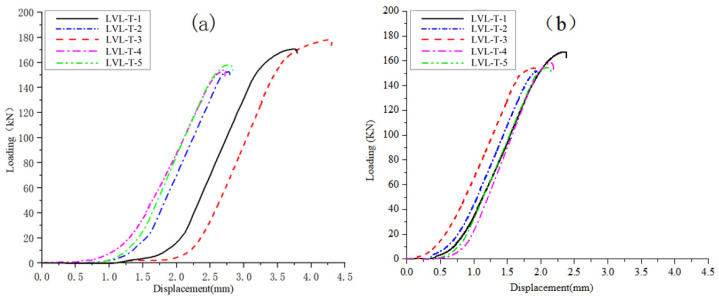
Load–displacement curve of longitudinal compressive strength. (**a**) LVL-CF specimen longitudinal compression test load–displacement curve. (**b**) LVL-T specimen longitudinal compression test load–displacement curve.

The load–displacement curves of the longitudinal compression tests for LVL-CF (a) and LVL-T (b) specimens are shown in Figure 10, and the longitudinal compression test data are presented in Table 8 and Table 9. According to the data in the table, the characteristic value of the longitudinal compressive strength of a is 54.32 MPa, which meets the standard 15 × 10^−45^; the characteristic value of the longitudinal compressive strength of b is 52.36 MPa, which also meets the standard 15 × 10^−45^. The longitudinal compressive strength of a is 1.04 times that of b.

According to Figure 10, during the initial loading stage, the specimen is in the elastic working stage, and the curve shows a linear growth state. As the load continues to increase, the slope of the curve gradually shifts. When the test piece reached the failure load, it made a loud noise, was crushed, and some adhesive layers cracked. However, the test piece could continue to bear the load, indicating that the LVL test piece had ductile failure in terms of compressive strength along the grain. According to the analysis of the load–displacement curve, it was found that the compressive failure curves of each specimen of a along the grain are highly divergent, and the displacement values increase too quickly in the early stage of loading. This is due to the presence of gaps at the internal joints of LVL, which gradually become cracks during the compression process along the grain, thereby affecting its performance indicators. In summary, carbon fiber is not significantly effective in enhancing the longitudinal compressive performance of LVL beams in timber structures.

#### 3.3.6. Results and Analysis of Transverse Parallel Compression Test

**Figure 11:**
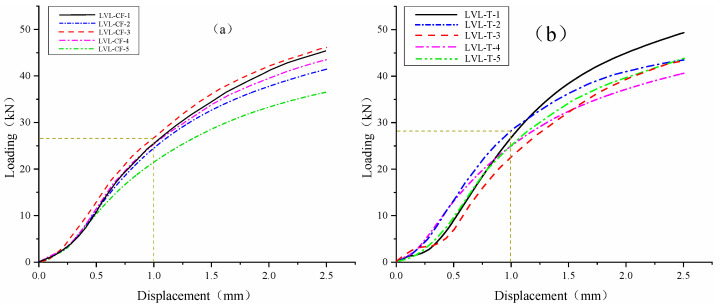
Horizontal parallel compressive strength load–displacement curve. (**a**) LVL-CF specimen transverse parallel compressive load–displacement curve. (**b**) LVL-T specimen transverse parallel compressive load–displacement curve.

The load–displacement curves of the transverse parallel compression test for LVL-CF (a) and LVL-T (b) specimens are shown in Figure 11. The load–displacement curves of the transverse compression specimens of a and b are basically consistent and have a high degree of overlap.

The parallel compression test data of horizontal stripes are shown in Table 10 and Table 11. These are assessed according to the performance indicators achieved in the standard GB/T 17657-2013, “Test Methods for Physical and Chemical Properties of Artificial Boards and Decorative Artificial Boards”. From the table, it can be seen that the average transverse parallel compressive strength of a is 10.06 MPa, which meets the standard 15 × 10^−45^; the average transverse parallel compressive strength of b is 9.76 MPa, which meets the standard 14 × 10^−42^. The transverse parallel compressive strength of a is 1.03 times that of b.

#### 3.3.7. Results and Analysis of Parallel Shear Test Along the Grain

**Figure 12:**
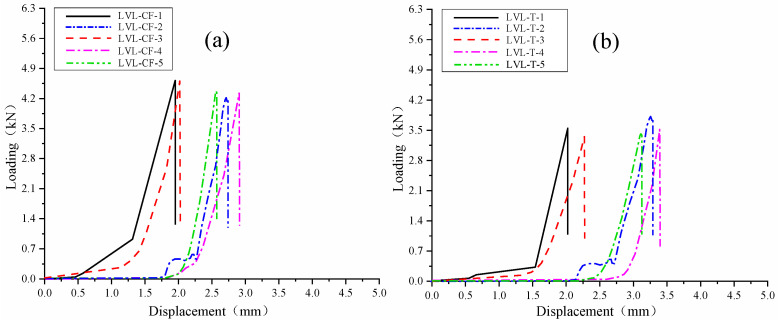
Load–displacement curve of parallel shear strength along the grain. (**a**) LVL-CF specimen parallel shear test load–displacement curve. (**b**) LVL-T specimen parallel shear test load–displacement curve.

The load–displacement curves of parallel shear tests along the grain for LVL-CF (a) and LVL-T (b) specimens are shown in Figure 12, and the data of parallel shear tests along the grain are shown in Table 12 and Table 13. From the data in the table, it can be calculated that the characteristic value of the parallel shear mechanical properties of a is 6.49 MPa, which meets the standard 15 × 10^−45^; the characteristic value of the parallel shear strength of b is 5.44 MPa, which meets the standard 14 × 10^−42^. The parallel shear strength of a is 1.2 times that of b.

## 4. Conclusions

In this study, carbon-fiber-reinforced laminated lumber (LVL) beams based on lignin from corn stover were successfully developed for garden building structures, which effectively solved the wood shortage and environmental problems. The results showed that the lignin extracted by deep eutectic solvent (DES) has high molecular weight and homogeneity, which is suitable for carbon fiber preparation. The prepared lignin-based carbon fibers exhibited the best mechanical properties at a lignin-to-polyacrylonitrile (PAN) ratio of 20:80, with tensile strength and Young’s modulus reaching 30.4 MPa and 4.8 GPa, respectively, which were close to those of conventional PAN-based carbon fibers, while reducing the cost. The reinforced LVL beams showed a 17% increase in the modulus of elasticity and a 30% increase in flexural strength, and exhibited significant improvements in tensile, compressive, and shear strength. In addition, the preparation of high-value-added carbon fibers from agricultural waste is in line with the goal of sustainable development, reduces environmental impact, and promotes resource recycling. Future research will focus on optimizing the hot-pressing process parameters of LVL beams, improving the extraction process of high molecular weight lignin, developing more environmentally friendly polymer blending systems, and expanding the application areas of lignin-based carbon-fiber-reinforced composites in order to promote the sustainable development of wood structures.

## Figures and Tables

**Table 1:** Results of lignin molecular weight determination [30].

Sample	M_N_	M_W_	PD (M_W_/M_N_)
ChCl-EG	36,837	45,156	1.2
ChCl-H	38,229	46,294	1.2
ZnCl_2_-EG	24,019	36,029	1.5
Alkali Lignin	791	2408	3.1

**Table 2:** Experimental data on moisture content.

Number	Size (mm × mm × mm)	Initial Mass m_w_ (G)	Drying Quality m^0^ (G)	Moisture Content W (%)
1	50 × 50 × 50	102.4	91.43	12.0
2	50 × 50 × 50	103.5	92.01	12.5
3	50 × 50 × 50	101.9	91.23	11.7
4	50 × 50 × 50	103.1	91.32	12.9
5	50 × 50 × 50	102.5	91.03	12.6
6	50 × 50 × 50	103.9	92.21	12.4
Average	-			12.3

**Table 3:** Immersion and peel test data.

Number	Layered Length/mm	Size	Layered Length/mm
1	7	6	9
2	9	7	13
3	11	8	8
4	6	9	12
5	10	10	10

**Table 4:** LVL-CF (a): bending strength and elastic modulus test data.

Number	Failure Load (kN)	Bending Strength (MPa)	Elastic Modulus (MPa)	Standard Deviation (MPa)
1	8.16 × 10^3^	58.75	14,224.0	-
2	7.75 × 10^3^	55.80	13,805.9	-
3	7.54 × 10^3^	54.29	13,581.9	-
4	7.62 × 10^3^	54.86	13,376.7	-
5	7.14 × 10^3^	51.41	13,137.6	-
Average	-	55.02	13,625.2	2.37

**Table 5:** LVL-T (b): bending strength and elastic modulus test data.

Number	Failure Load (kN)	Bending Strength (MPa)	Elastic Modulus (MPa)	Standard Deviation (MPa)
1	6.25 × 10^3^	45.02	12,386.4	-
2	5.77 × 10^3^	41.57	11,146.1	-
3	5.63 × 10^3^	40.55	10,679.4	-
4	6.02 × 10^3^	43.37	11,773.2	-
5	6.52 × 10^3^	46.97	12,426.7	-
Average	-	43.50	11,682.3	2.31

**Table 6:** LVL-CF (a): longitudinal tensile test data.

Number	Size (mm × mm × mm)	Failure Load (kN)	Longitudinal Compressive Strength (MPa)	Standard Deviation (MPa)
1	2100 × 50 × 50	169.10	67.64	-
2	2100 × 50 × 50	164.05	65.62	-
3	2100 × 50 × 50	171.33	68.53	-
4	2100 × 50 × 50	168.84	67.54	-
5	2100 × 50 × 50	167.91	67.16	-
Average	-		67.30	0.95

**Table 7:** LVL-T (b): longitudinal tensile test data.

Number	Size (mm × mm × mm)	Failure Load (kN)	Longitudinal Compressive Strength (MPa)	Standard Deviation (MPa)
1	2100 × 50 × 50	112.08	44.83	-
2	2100 × 50 × 50	113.95	45.58	-
3	2100 × 50 × 50	114.45	45.78	-
4	2100 × 50 × 50	106.73	42.69	-
5	2100 × 50 × 50	117.02	46.80	-
Average	-		45.14	1.38

**Table 8:** LVL-CF (a): longitudinal compression test data.

Number	Size (mm × mm × mm)	Failure Load (kN)	Longitudinal Compressive Strength (MPa)	Standard Deviation (MPa)
1	125 × 50 × 50	168.87	67.55	-
2	125 × 50 × 50	151.54	60.62	-
3	125 × 50 × 50	178.04	71.22	-
4	125 × 50 × 50	152.31	60.92	-
5	125 × 50 × 50	156.25	62.50	-
Average	-		64.56	4.15

**Table 9:** LVL-T (b): longitudinal compression test data.

Number	Size (mm × mm × mm)	Failure Load (kN)	Longitudinal Compressive Strength (MPa)	Standard Deviation (MPa)
1	125 × 50 × 50	164.81	65.92	-
2	125 × 50 × 50	150.93	60.37	-
3	125 × 50 × 50	153.02	61.21	-
4	125 × 50 × 50	156.87	62.75	-
5	125 × 50 × 50	152.78	61.11	-
Average	-		62.27	1.98

**Table 10:** LVL-CF (a): horizontal parallel compressive test data.

Number	Size (mm × mm × mm)	1.00 mm Deformation Load (kN)	Transverse Compressive Strength (MPa)	Standard Deviation (MPa)
1	150 × 50 × 50	26.25	10.50	-
2	150 × 50 × 50	27.31	10.92	-
3	150 × 50 × 50	22.51	9.01	-
4	150 × 50 × 50	24.67	9.87	-
5	150 × 50 × 50	25.05	10.02	-
Average	-		10.06	0.64

**Table 11:** LVL-T (b): horizontal parallel compressive test data.

Number	Size (mm × mm × mm)	1.00 mm Deformation Load (kN)	Transverse Compressive Strength (MPa)	Standard Deviation (MPa)
1	150 × 50 × 50	25.11	10.04	-
2	150 × 50 × 50	24.02	9.61	-
3	150 × 50 × 50	25.98	10.39	-
4	150 × 50 × 50	24.97	9.99	-
5	150 × 50 × 50	21.87	8.75	-
Average	-		9.76	0.56

**Table 12:** Experimental data on parallel shear resistance ofLVL-CF (a).

Number	Size (mm × mm × mm)	Failing Load (kN)	Parallel Shear Strength of Smooth Fiber (MPa)	Standard Deviation (MPa)
1	50 × 20	4.60	7.30	-
2	50 × 20	4.58	7.10	-
3	50 ×20	4.36	6.91	-
4	50 × 20	4.17	6.71	-
5	50 ×20	4.29	6.89	-
Average	-		6.98	0.20

**Table 13:** Experimental data on parallel shear resistance ofLVL-T (b).

Number	Size (mm × mm × mm)	Failing Load (kN)	Parallel Shear Strength of Smooth Fiber (MPa)	Standard Deviation (MPa)
1	50 × 20	3.58	6.23	-
2	50 × 20	3.79	6.31	-
3	50 ×20	3.29	5.89	-
4	50 × 20	3.50	6.24	-
5	50 ×20	3.42	6.19	-
Average	-		6.17	0.15

## Data Availability

Original contributions to this study are included in the article. For further inquiries, please contact the corresponding author directly.
